# The MADS-box gene *FveSEP3* plays essential roles in flower organogenesis and fruit development in woodland strawberry

**DOI:** 10.1038/s41438-021-00673-1

**Published:** 2021-12-01

**Authors:** Mengting Pi, Shaoqiang Hu, Laichao Cheng, Ruhan Zhong, Zhuoying Cai, Zhongchi Liu, Jia-Long Yao, Chunying Kang

**Affiliations:** 1 Key Laboratory of Horticultural Plant Biology (Ministry of Education), Hubei Hongshan Laboratory, Huazhong Agricultural University, Wuhan, China; 2 Department of Cell Biology and Molecular Genetics, University of Maryland, College Park, MD USA; 3 The New Zealand Institute for Plant and Food Research Ltd, Auckland, New Zealand

**Keywords:** Plant development, Plant reproduction

## Abstract

Flower and fruit development are two key steps for plant reproduction. The ABCE model for flower development has been well established in model plant species; however, the functions of ABCE genes in fruit crops are less understood. In this work, we identified an EMS mutant named R27 in woodland strawberry (*Fragaria vesca*), showing the conversion of petals, stamens, and carpels to sepaloid organs in a semidominant inheritance fashion. Mapping by sequencing revealed that the class *E* gene homolog *FveSEP3* (FvH4_4g23530) possessed the causative mutation in R27 due to a G to E amino acid change in the conserved MADS domain. Additional *fvesep3*^*CR*^ mutants generated by CRISPR/Cas9 displayed similar phenotypes to *fvesep3-R27*. Overexpressing wild-type or mutated *FveSEP3* in Arabidopsis suggested that the mutation in R27 might cause a dominant-negative effect. Further analyses indicated that FveSEP3 physically interacted with each of the ABCE proteins in strawberry. Moreover, both R27 and *fvesep3*^*CR*^ mutants exhibited parthenocarpic fruit growth and delayed fruit ripening. Transcriptome analysis revealed that both common and specific differentially expressed genes were identified in young fruit at 6–7 days post anthesis (DPA) of *fvesep3* and pollinated wild type when compared to unpollinated wild type, especially those in the auxin pathway, a key hormone regulating fruit set in strawberry. Together, we provided compelling evidence that *FveSEP3* plays predominant E functions compared to other E gene homologs in flower development and that *FveSEP3* represses fruit growth in the absence of pollination and promotes fruit ripening in strawberry.

## Introduction

The octoploid strawberry species *Fragaria* × *ananassa* is cultivated worldwide as an economically important fruit crop. The diploid woodland strawberry *Fragaria vesca* has emerged as a model species for research on flower development and fruit ripening^1,2^. The flower is an essential organ in angiosperms for fruit production. *F. vesca* flowers have four typical whorls of floral organs, namely, sepals, petals, stamens, and carpels. A special feature of strawberry flowers is that numerous carpels are independently developed and attached to the receptacle, which is enlarged to generate juicy flesh, while dry achenes are botanical true fruits^3^. Given the importance of flowers, the developmental control of strawberry flowers remains unclear.

To date, the well-known ABCE model for flower development explains the basic tenets of floral organ specification. In this model, four classes of homeotic genes (A, B, C, and E) work coordinately to determine the identity of each whorl of floral organs. Specifically, class A genes (*APETALA1*, *AP1*; *APETALA2*, *AP2*) specify sepal identity; class A and B (*APETALA3*, *AP3*; *PISTILLATA*, *PI*) genes specify petal identity; class B and C (*AGAMOUS*, *AG*) genes specify stamen identity; class C genes specify carpel identity; and class E genes (*SEPALLATA*, *SEP*) are required for floral organ determination in all four whorls^4^. ABCE proteins can interact physically with each other to form higher-order protein complexes and bind to the CArG-box motifs of downstream genes to regulate their transcription^5–8^.

Almost all ABCE genes encode MIKC-type MADS-box transcription factors, with the only exception being *AP2*. These proteins consist of a highly conserved MADS domain for DNA binding, a K domain for protein interaction, and a less conserved intervening I region and C-terminal region^9^. Class E genes form a small family in different plant species. For instance, there are four *SEP* members in Arabidopsis, namely, *SEP1*–*4*. Their single mutants exhibited subtle phenotypes, but the floral organs could be converted into sepals or leaves in the triple or quadruple mutants^10,11^, indicating that they play largely redundant roles in flower development.

After pollination, fruits usually develop from ovarian tissues, such as tomato (*Solanum lycopersicum*), or occasionally from nonovary floral tissues, such as the receptacle in strawberry or the hypanthium in apple (*Malus domestica*). Parthenocarpy is defined as fruit development without fertilization, which is a desired trait to ensure high yields under unfavorable conditions for pollination. In strawberry, the hormones auxin and gibberellic acid (GA) play important roles in parthenocarpy^12,13^. In addition, homeotic genes in flower development also take part in controlling fruit development. For example, reduced expression of either *TM29* (*SEP1/2*-like) or *TM5* (*SEP3*-like) caused parthenocarpy in tomato^14,15^. The suppression of *MdMADS8/9* (*SEP1/2*-like) in apple led to a loss of flesh tissue developed from hypanthium and a delay in ripening^16^. Some apple varieties develop parthenocarpic fruit owing to transposon insertion in the introns of *MdPI*^17^. These results indicate that the class B and E genes act as repressors of fruit development when fertilization does not occur.

In *F. vesca*, the class ABCE genes have been identified according to sequence homology, and their expression patterns exhibit tissue specificity in floral organs, consistent with the supposed regulatory functions during flower development^18^. However, whether they truly play ABCE functions is still unknown. The knockout of *FaTM6* in cultivated strawberry, the ortholog of *FveAP3*, caused the defective development of petals and stamens^19^. The suppression of *FaMADS9* (*SEP1/2*-like) resulted in the green coloration of petals and delayed ripening^20^. Other ABCE gene homologs have not been functionally studied in strawberry.

In this work, we identified an EMS mutant in *F. vesca* called R27, which developed sepaloid floral organs in the whorls of petals, stamens, and carpels. Additionally, R27 developed fruit without fertilization. Gene isolation revealed a point mutation in *FveSEP3* causing a single amino acid G to E conversion in the MADS domain. The identification of the R27 mutant allowed us to unveil the functions of *FveSEP3* in flower and fruit development in woodland strawberry.

## Results

### EMS mutant R27 in *F. vesca* developed sepaloid floral organs in the inner three whorls

**Fig. 1:**
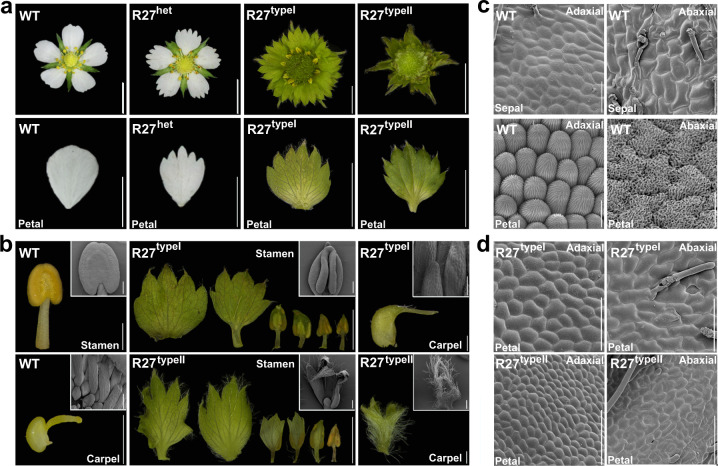
Phenotypes of the *Fragaria vesca* EMS mutant R27. **a** Images showing whole flowers and petals of wild-type (WT, Ruegen) and heterozygous and homozygous R27 mutants. Homozygous R27 flowers were further classified into type I and type II. **b** Images showing the stamens and carpels of WT and R27. Insets are scanning electron microscopic (SEM) images. **c** SEM images showing the adaxial and abaxial epidermis of WT sepals and petals. **d** SEM images showing the adaxial and abaxial epidermis of R27 petals. Scale bars: **a** 0.5 cm; **b** 1 mm for stamens, 0.5 mm for carpels, 200 μm for SEM images; **c**, **d** 10 μm for petals, 50 μm for sepals

The flowers of woodland strawberry *F. vesca* contain four typical whorls of organs, including 5 sepals, 5 white petals, 20 stamens, and numerous unfused carpels attached to the domed receptacle^3^. To discover essential genes regulating flower morphogenesis, we screened the EMS mutagenized M_2_ population of the *F. vesca* variety Ruegen generated as described previously^21^ and identified a mutant numbered R27 with defective floral organs (Fig. 1a). Compared to the wild type (WT), the heterozygous R27 developed basically normal flowers, except that the petals had serrated margins. The homozygous R27 mutant fell into two types based on the severity of floral defects. A majority of R27 had type I flowers, and only a small fraction developed type II flowers. Both types of flowers had green leaf-like organs instead of white petals (Fig. 1a). However, the third and fourth whorls exhibited more diverse phenotypes. In type I flowers, the outer stamens became green and flat tissues with serrated margins; the inner stamens still developed into filaments and anthers, but the anthers had deeper dents between the yellow locules and contained green mosaic tissues (Fig. 1b). The carpels of type I flowers had a similar shape to the WT carpels, but the carpel walls became green instead of pale green, and the styles were more pointy (Fig. 1b). In type II flowers, a higher percentage of stamens became green leaf-like tissues with more epidermal hairs, and the carpels completely lost the regular structure and became leaf-like tissues with dense epidermal hairs (Fig. 1b).

To determine the identity of the sepaloid tissues, scanning electron microscopy (SEM) was used to examine the epidermal cells. In the WT, the adaxial and abaxial sides of sepals had epidermal cells with irregular size and shape; additionally, the abaxial side bore stomata and epidermal hairs (Fig. 1c). The adaxial side of petals in the WT had conical cells with straight epicuticular ridges running from the apex to the base, while the abaxial epidermal cells had irregular shapes with wavy epicuticular ridges (Fig. 1c). In contrast, the green tissues converted from petals in type I and II flowers completely lost the features of petal epidermal cells and were similar to sepals on both adaxial and abaxial sides (Fig. 1d). Moreover, type II flowers exhibited internode elongation between the stamen whorl and carpel whorl (Supplementary Fig. S1), suggesting that the entire flower was partially converted into a shoot.

### Floral organ morphologies at different developmental stages in R27

**Fig. 2:**
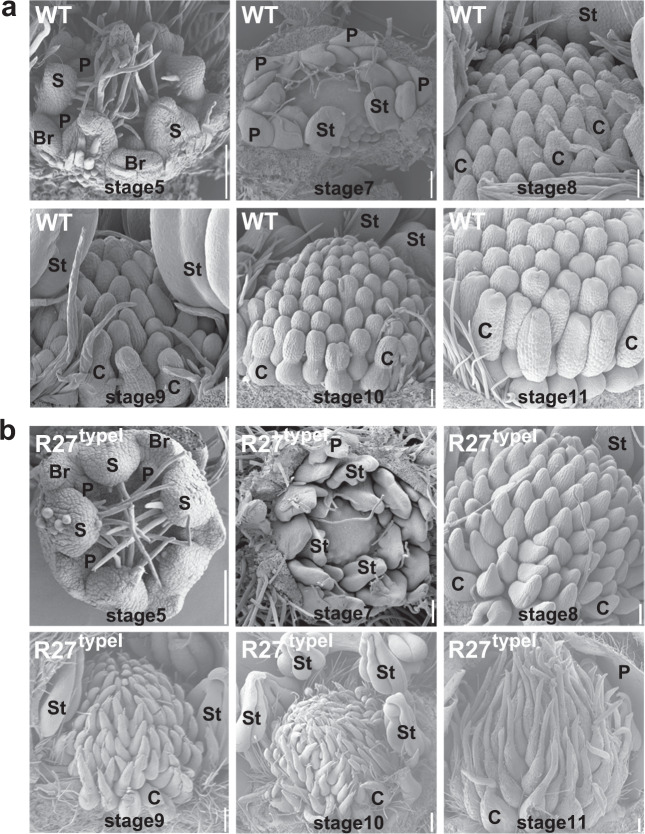
Morphologies of wild-type and R27-type I floral buds at different developmental stages. SEM images showing the floral buds of wild type (**a**) and R27 (**b**) at developmental stages 5–11. The sepals and petals were removed to expose stamens and carpels starting from stage 7. S, sepal; Br, bract; P, petal; St, stamen; C, carpel. Scale bars: **a** 0.5 mm; **b** 0.5 mm for stages 5–8, 1 mm for stages 9–11

To examine the development process of floral organs, flowers of WT and R27 were further observed at different stages by SEM. Only type I flowers in R27 were examined, as they were the main type. At stage 5, the five sepals appeared with long epidermal hairs at the adaxial side, and petal primordia were initiated (Fig. 2). There was no obvious morphology difference between the WT and R27 flowers up to this stage. At stage 7, the petals in the WT arose with smooth margins, the stamens exhibited a slightly lobed structure, and the carpel primordia initiated from the receptacle dome acropetally. In R27, the petals exhibited three serrations with epidermal hairs engendering at the abaxial surface, and the stamens showed the emergence of protrusions at the adaxial side (Fig. 2). At stage 8, all the carpels arose without obvious differences in shape between the WT and R27. At stage 9, the WT carpels exhibited a water prop-like shape with round tips, while the R27 carpels had thin and pointy styles with epidermal hairs emerging. At stages 10 and 11, the WT carpels possessed bifurcated style atop without any hairs, while the pointy styles on the top of R27 carpels grew longer and bore more and longer epidermal hairs (Fig. 2).

### R27 was caused by a G to E amino acid mutation in FveSEP3

**Fig. 3:**
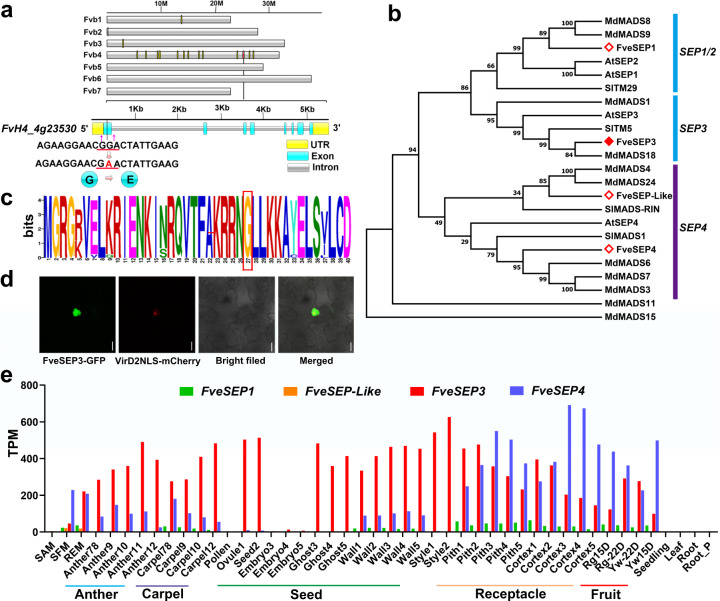
Identification and characterization of *FveSEP3*. **a** Diagram showing the locations of high-quality exonic SNPs. The red line indicates the candidate causative SNP in FvH4_4g23530 (*FveSEP3*) that results in the conversion of G to A in DNA and G (Gly) to E (Glu) in protein. The arrows indicate the positions of primers used for examining the mutation. **b** Phylogenetic tree of the SEP proteins of *F. vesca*, Arabidopsis, tomato, and apple using full-length sequences. **c** Sequence logo of the first 40 amino acids in the MADS domain based on 18 homologs showing the conserved amino acids. The relative heights of letters indicate the frequencies that appeared in those sequences. **d** Subcellular localization of FveSEP3-GFP in tobacco leaves. Green indicates GFP fluorescence, and red indicates mCherry fluorescence from the nuclear marker VirD2NLS-mCherry. Scale bars: 50 μm. **e** Expression levels (TPMs) of the four *FveSEP* genes in *F*. *vesca* according to the RNA-seq analysis

The R27 mutant was found in the M_2_ EMS population of the *F. vesca* variety Ruegen. To identify the causative mutation, the heterozygous R27 mutant was backcrossed with WT Ruegen to segregate away unrelated background mutations. The heterozygous R27 F1 from the backcross was selfed to generate an F_2_ population, in which a segregation ratio of 23:47:22 (WT:heterozygous:homozygous) was observed. This ratio was fitted to 1:2:1 (*χ*^2^ = 1.875; *χ*^2^_0.05_ = 5.99), suggesting that a single gene accounted for the striking phenotypes. Young leaves of 20 heterozygous mutants and 20 homozygous mutants were pooled for whole-genome sequencing. Totals of 28.7 million and 30.5 million paired-end reads at 150 bp were obtained for the two groups. Bioinformatics analysis revealed 19 high-quality single-nucleotide polymorphisms (SNPs) after filtering based on our in-house pipeline. Of these SNPs, 18 were located in the exonic regions on chromosome 4 (Table S1). After filtering, the G to A SNP located in the first exon of FvH4_4g23530, causing amino acid conversion from G (Gly) to E (Glu) at residue position 27, was considered the primary candidate (Fig. 3a). This mutation was further confirmed to be homozygous in 44 F_2_ mutants by polymerase chain reaction (PCR) amplification and Sanger sequencing of the amplified DNA. Sequence analysis revealed that FvH4_4g23530 shared a high similarity to the MADS-box gene *SEP3* in Arabidopsis, belonging to the class E floral homeotic genes^10,11,22^. Therefore, this gene was named *FveSEP3* as previously reported^18^, and R27 is called *fvesep3* hereafter.

### Characterization of *FveSEP3* and its homologs

To better understand the gene functions, a phylogenetic tree was constructed using the homologs of FveSEP3 in strawberry, Arabidopsis, and tomato (*S. lycopersicum*). These proteins could be divided into three clades: the SEP1/2 clade, including FveSEP1; the SEP3 clade, including FveSEP3; and the SEP4 clade, including FveSEP4 and FveSEP-Like (Fig. 3b). Sequence alignment revealed that all the FveSEPs had conserved MADS domains and K domains in the typical MIKC-type MADS-box protein (Supplementary Fig. S2). Moreover, their homologs in the cultivated strawberry genome were also identified^23,24^. There were two *FveSEP1* homologs (FxaC_21g10230, FxaC_22g08610), three *FveSEP3* homologs (FxaC_13g22210, FxaC_15g15520, FxaC_14g14650), and three *FveSEP4* homologs (FxaC_17g18090, FxaC_20g14200, FxaC_18g32540) (Supplementary Fig. S2a). The mutation in FveSEP3 was located in the highly conserved MADS domain, as indicated by sequence alignment of 18 FveSEP3 homologs in different plant species (Fig. 3c).

To detect the subcellular localization of FveSEP3, the *3S::FveSEP3-GFP* construct was transiently transformed into tobacco leaves mediated by agroinfiltration. The results showed that the FveSEP3-GFP signal was detected in the nucleus and colocalized with the nuclear marker VirD2NLS-mCherry^25^, consistent with its roles in the regulation of transcription (Fig. 3d). Moreover, the expression patterns of the four *FveSEP* genes were obtained from the transcriptome database generated from a range of vegetative and reproductive tissues of *F. vesca*^26^. The results demonstrated that *FveSEP3* was broadly expressed not only in the floral tissues, such as anthers and carpels, but also in the fruit, including the ghosts (endosperms and seed coats), carpel walls, and especially receptacles (cortex and pith) (Fig. 3e). *FveSEP4* was expressed at low levels in flowers and seeds but was more highly expressed in receptacles than *FveSEP3*. In contrast, *FveSEP1* and *FveSEP-Like* were expressed at much lower levels. According to RNA sequencing (RNA-seq) data generated from cultivated strawberry, the three *FveSEP3* homologs were also most abundantly expressed in both achenes and fruit receptacles compared to other genes (Supplementary Fig. S2b). These expression patterns indicated that *FveSEP3* and *FveSEP4* might play important roles in both flowers and fruit in strawberry.

### The *fvesep3*^*CR*^ mutants exhibited phenotypes similar to *fvesep3*

**Fig. 4:**
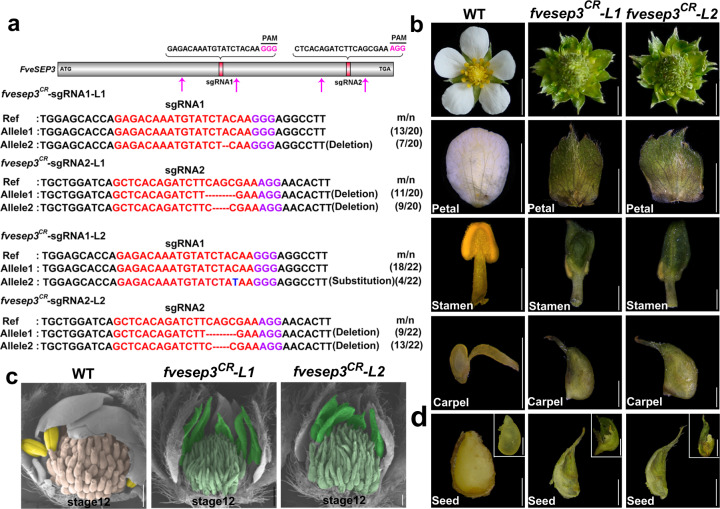
Generation and characterization of *fvesep3*^*CR*^ mutants in *Fragaria vesca*. **a** The top panel shows the location and sequences of two guide RNAs (sgRNA1 and sgRNA2) used to generate mutations in the *FveSEP3* gene. The sgRNA target sites in two T_0_ mutant lines, *fvesep3*^*CR*^ L1 and L2, were analyzed to show sequence variations examined with the primers indicated by magenta arrows. The sgRNA is indicated with red. The mutated nucleotides are indicated with blue, and deletions are indicated with dashes. For *m*/*n*, *n* indicates the number of clones examined, and *m* indicates the number of bacterial colonies showing the indicated sequence. **b** Images showing the whole flowers and dissected floral organs of the wild-type (WT) *F. vesca* variety H4 and its two gene-edited lines *fvesep3*^*CR*^ L1 and L2. **c** SEM images showing the stage 12 flowers of WT and *fvesep3*^*CR*^ L1 and L2. Stamens and carpels are false-colored. **d** Images showing the seeds and embryos of WT and *fvesep3*^*CR*^ L1 and L2. Scale bars: **b** 0.5 cm for flowers and petals, 1 mm for stamens and carpels; **c** 5 mm; **d** 1 mm for seeds, 0.5 mm for embryos

R27/*fvesep3* exhibited a semidominant inheritance habit, making it hard to define gene functions. To clarify this question, clustered regularly interspaced short palindromic repeats/CRISPR-associated protein 9 (CRISPR/Cas9)-mediated genome editing was performed to knock out *FveSEP3* in the *F. vesca* strain Hawaii 4 (H4). Two *FveSEP3*-specific single-guide RNA (sgRNA) target sites were designed in one construct. A total of ten independent transgenic lines were obtained and confirmed by the amplification of the *Cas9* and *GFP* fragments (Supplementary Fig. S3). All of them developed aberrant flowers in the T_0_ generation, indicating a high editing efficiency. Then we examined the induced mutations at the two target sites by cloning and Sanger sequencing in the two lines. In Line 1, a proportion of the clones from the sgRNA1 target site harbored a 1-bp deletion, while all the clones from sgRNA2 harbored either 2- or 4-bp deletions (Fig. 4a). In Line 2, the sgRNA1 target site harbored both point mutation (C to T) and WT alleles, but all of the clones from the sgRNA2 target site were mutated with short deletions. These mutations resulted in frameshift and truncated FveSEP3 in both *fvesep3*^*CR*^ lines. No editing was found in the other three *FveSEP* genes by sequencing. Furthermore, the expression level of the *FveSEP3*^*CR*^ mutant transcripts was greatly reduced in the *fvesep3*^*CR*^ mutant lines (Supplementary Fig. S4), suggesting that *FveSEP3* might be null in these lines.

The two *fvesep3*^*CR*^ mutant lines exhibited similar alterations in flowers to each other and to type I *fvesep3*. Specifically, the petals and stamens became sepaloid organs (Fig. 4b). The carpels were similar to WT, consisting of two parts, but the styles became shorter and thinner with dried tips. Closer observation revealed a striking similarity between *fvesep3*^*CR*^ and type I *fvesep3* at stage 12 flowers by SEM, such as the pointy and hairy carpels (Fig. 4c). Dissection of the achenes at 30 days post anthesis (DPA) revealed no seeds inside, suggesting failure in fertilization (Fig. 4d). These results supported that *FveSEP3* plays predominant roles in floral organogenesis among the E genes.

### The floral homeotic genes were differentially expressed in *fvesep3*

**Fig. 5:**
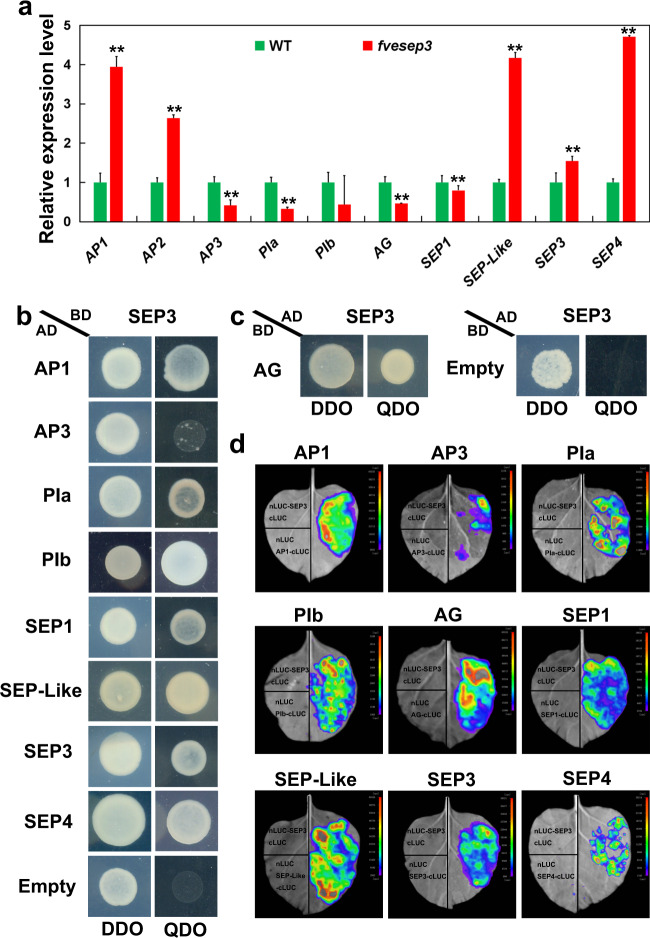
Interactions between FveSEP3 and the ABCE class of MADS-box proteins. **a** Relative expression levels of the class ABCE genes in stage 9–10 flowers of wild-type and R27/*fvesep3* strawberry plants were determined using qRT-PCR analysis. Data are the means ± SD obtained from three biological replicates. **, *P* < 0.01, Student’s *t* test. **b**, **c** Physical interactions between FveSEP3 and class ABCE MADS-box proteins in strawberry were examined by yeast two-hybrid assay. Transformed yeast cells were grown on SD-Leu-Trp (DDO) and SD-Leu-Trp-His-Ade (QDO). AD, activation domain, BD, DNA-binding domain. Empty vectors were used as controls. **d** Physical interactions between FveSEP3 and class ABCE MADS-box proteins (labeled below) were examined by split luciferase assay. The genes were transiently expressed in tobacco leaves. On the left are the negative controls. On the right are the interaction signals between nLUC-SEP3 and A/B/C/E-cLUC

In Arabidopsis, SEP3 can directly bind to the promoters of thousands of downstream genes to regulate their expression levels, including ABCE floral homeotic genes^27^. To compare the expression levels of these genes, flower buds at stages 9–10 of WT and *fvesep3* were used for quantitative reverse transcriptase (qRT)-PCR. The two class A genes, *FveAP1* and *FveAP2*, were significantly increased in *fvesep3*, while the class B and C genes (*FveAP3*, *FvePIa*, and *FveAG*) were downregulated in *fvesep3* (Fig. 5a), which is consistent with the conversion of petals and stamens into sepaloid organs and leaf-like carpels. The *FveSEP3* transcript itself is significantly upregulated in *fvesep3*, perhaps due to feedback regulation at the transcription level. This might result in high levels of the FveSEP3^G27E^ mutant protein in *fvesep3*. Interestingly, two class E genes (*FveSEP-Like* and *FveSEP4*) were also dramatically upregulated in *fvesep3* (Fig. 5a), suggesting either direct transcriptional regulation or a transcriptional compensation effect.

### FveSEP3 physically interacts with the ABCE class of MADS-box proteins

The class ABCE proteins could physically interact with each other to form higher-order protein complexes^5,6^. To investigate whether strawberry ABCE class MADS-box proteins interact among themselves, a yeast two-hybrid assay was performed. The results showed that FveSEP3 strongly interacted with all ABCE class MADS-box proteins except FveAP3 (Fig. 5b, c and Supplementary Fig. S6a). These interactions were also verified by the split-luciferase assay (Fig. 5d). The fluorescence intensity in the combination of FveSEP3 and FveAP3 was much lower than that in the other combinations, indicating that they interacted with each other more weakly. These results suggested that the strawberry SEP3 protein is able to interact with the A, B, C, and E classes of proteins.

### *FveSEP3*^*G27E*^ overexpression caused sepaloid floral organs in Arabidopsis

Genetic studies revealed that the *fvesep3*/R27 mutant exhibited a semidominant inheritance habit. To determine the functions of *FveSEP3*^*G27E*^, WT *FveSEP3* and *FveSEP3*^*G27E*^ were overexpressed, driven by the 35S promoter in WT Arabidopsis. Twenty independent *FveSEP3-ox* transgenic lines were obtained in the T_1_ generation, all of which looked like the WT. For *FveSEP3*^*G27E*^ overexpression, 15 out of 44 transgenic lines (34%) exhibited defective floral organs in the T_1_ generation (Supplementary Fig. S5a). Compared to the two *FveSEP3-ox* lines (L19 and L15), *FveSEP3*^*G27E*^ was expressed at a similar level in L1 and a much lower expression level in L2, as examined by qRT-PCR (Supplementary Fig. S5b). Close observation revealed that the sepals of *FveSEP3*^*G27E*^*-ox* L1 became leaf-like organs and much larger in size, that the petals and stamens became sepaloid organs, and that the gynoecium was replaced by a stalked carpel (Supplementary Fig. S5c). These results indicated that the flower defects in *FveSEP3*^*G27E*^*-ox* were not caused by higher expression levels compared to *FveSEP3-ox*.

The *FveSEP3*^*G27E*^*-ox* phenotype resembled that of the *sep1/2/3* mutants in Arabidopsis^10,11^, indicating dysfunction in the Arabidopsis *SEP*s because of the introduction of *FveSEP3*^*G27E*^. To test the underlying mechanism, we examined the interactions between FveSEP3^G27E^ and other ABCE class MADS-box proteins by yeast two-hybrid assay. Similar to FveSEP3, FveSEP3^G27E^ could also interact with strawberry ABCE class MADS-box proteins (Supplementary Fig. S6a, b), which is in line with the intact K domain responsible for interaction. In contrast, FveSEP3^CR^ lost its interaction capacity with FvePIb and showed reduced interaction with FveSEP1 and FveSEP4 (Supplementary Fig. S6c). Therefore, we speculated that *FveSEP3*^*G27E*^ might be a dominant-negative allele.

### *FveSEP3* mutations caused parthenocarpic fruit and delayed fruit ripening

**Fig. 6:**
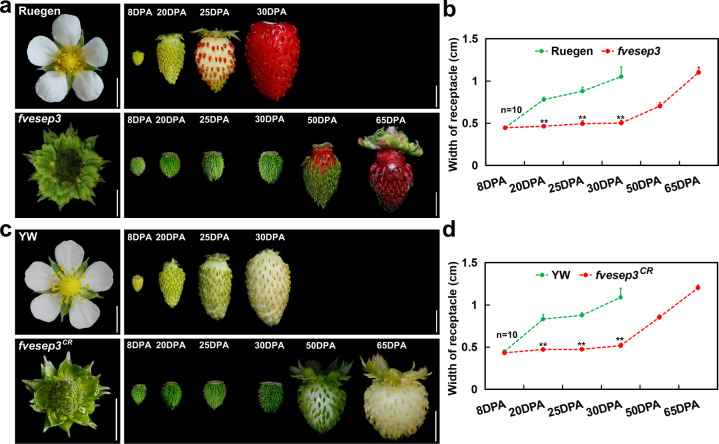
Fruit development of wild-type and *fvesep3* mutant strawberry plants. **a** Flowers at anthesis and fruits at different days post anthesis (DPA) of wild-type (red-fruited Ruegen) and *fvesep3*. **b** Width of receptacle at stages shown in **a**. **c** Flowers at anthesis and fruits at different days post anthesis (DPA) of wild-type (white-fruited YW) and *fvesep3*^*CR*^. **d** Width of receptacle at stages shown in **c**. In **b**, **d**, data are the means ± SD, *n* = 10; **, *P* < 0.01, Student’s *t* test

We noticed that both *fvesep3* and *fvesep3*^*CR*^ flowers exhibited receptacle enlargement, even though no fertilization occurred due to defective floral organs. To better characterize the fruit phenotype, fruit morphologies were observed from anthesis to full ripening in WT Ruegen and *fvesep3* (both red-fruited). The WT fruit enlarged gradually after fertilization, reached the turning stage at 25 DPA, and became mature at 30 DPA, as determined by the size and color (Fig. 6a). In contrast, the *fvesep3* fruit enlarged more slowly but finally ripened at 65 DPA, showing red pigments in both achenes and receptacles (Fig. 6a, b). Fruit growth was also observed in the *fvesep3*^*CR*^ mutants and the WT control YW (both white-fruited), showing similar trends to *fvesep3* (Fig. 6c, d). These results indicated that *FveSEP3* acts as a repressor of receptacle enlargement in the absence of fertilization and a promoter of fruit ripening.

### Functions of *FveSEP3* in fruit initiation

**Fig. 7:**
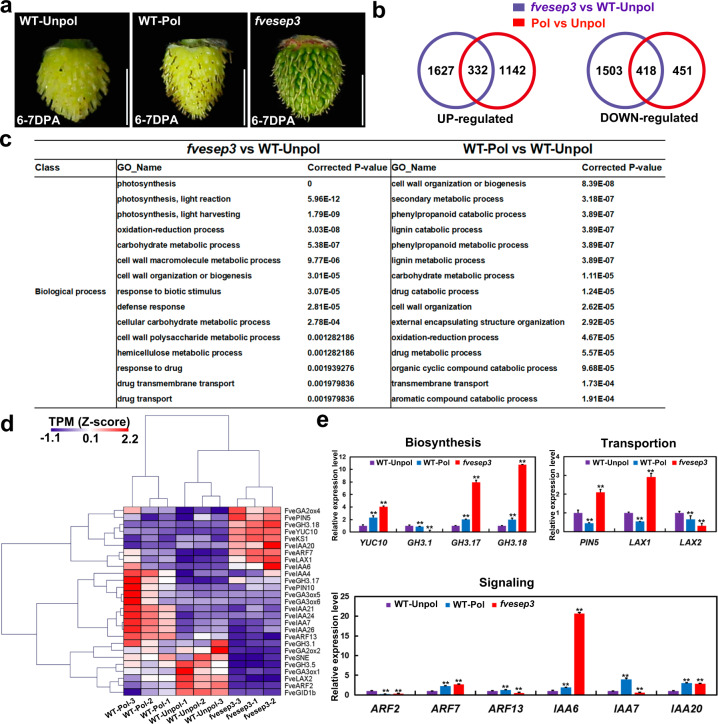
Transcriptome analysis of fruit at 6–7 DPA. **a** Fruit samples used for RNA-seq. **b** Venn diagram showing the differentially expressed genes in *fvesep3* and pollinated wild-type fruit compared to unpollinated wild-type fruit. **c** Top 15 enriched GO terms (corrected *P* value < 0.01) among the upregulated genes in *fvesep3* and pollinated wild-type fruit compared to unpollinated wild-type fruit. **d** Heatmap showing the expression levels of the differentially expressed auxin and GA pathway genes. The *Z*-score obtained from TPM values was used to present the expression levels. **e** Validation of the expression levels of the differentially expressed genes for auxin biosynthesis, transport, and signaling by qRT-PCR. Data are the means ± SD obtained from three biological replicates. **, *P* < 0.01, Student’s *t* test

To identify the roles of *FveSEP3* in fruit initiation, transcriptome analysis was performed for the entire young fruit (achenes and receptacle) at 6–7 DPA of *fvesep3*, the pollinated, and unpollinated WT fruit (Fig. 7a). Three biological replicates were harvested for each sample. Finally, a total of 29.4–43.4 million reads were obtained for each RNA-seq library with a high alignment percentage to the genome (>94%) (Table S2). Principal component analysis (PCA) revealed that the three biological replicates for each sample clustered closely together, while the samples from different materials were separated far away (Supplementary Fig. S7). Pairwise comparisons were carried out to identify differentially expressed genes. When *fvesep3* was compared to WT-Unpol, 1,959 genes were significantly upregulated, and 1,922 genes were significantly downregulated in *fvesep3* (*P* < 0.01, fold change >2); when WT-Pol was compared to WT-Unpol, 1,474 genes were significantly upregulated, and 869 genes were significantly downregulated (Data S1). Among these genes, there were 332 common upregulated genes and 418 common downregulated genes (Fig. 7b). Among the upregulated genes in *fvesep3* compared to WT-Unpol, the first three enriched gene ontology (GO) terms were related to photosynthesis, in line with the dark green color of *fvesep3* achenes (Fig. 7c). In both upregulated gene lists, there were also several common enriched GO terms on the cell wall and others, suggesting that similar biological processes were involved during *fvesep3* and pollinated WT fruit development.

Auxin and GA are the key hormones promoting fruit initiation in strawberry^13^. A total of 19 genes in the auxin pathway, including biosynthesis, transport, metabolism, and signaling, were differentially expressed in the pairwise comparisons (Table S3). Some of these genes were upregulated in both *fvesep3* and WT-Pol compared to WT-Unpol, such as *FveYUC10*, *FveLAX1*, *FveGH3.17*, and *FveARF7* (Fig. 7d). Some genes were upregulated in only one comparison; for example, *FveARF13*, *FveIAA4*, *FveIAA7*, *FveIAA21*, *FveIAA24*, and *FveIAA26* were specifically induced in WT-Pol, while *FvePIN5*, *FveGH3.18*, and *FveIAA20* were specifically induced in *fvesep3*. A small number of auxin genes were downregulated in both *fvesep3* and WT-Pol, such as *FveLAX2* and *FveARF2*. In addition, eight genes in the GA pathway were identified as differentially expressed genes, including the *FveGA3ox* and *FveGA2ox* genes responsible for GA biosynthesis and homeostasis (Fig. 7d). In the signaling pathway, the GA receptor gene *FveGID1b* was downregulated in both *fvesep3* and WT-Pol. To confirm the results, the expression levels of 13 auxin genes were examined by qRT-PCR in the same tissues used for RNA-seq analysis. A majority of the genes exhibited the same expression patterns as the RNA-seq data (Fig. 7e). Together, the *fvesep3* fruit employed similar pathways but sometimes different genes for growth in comparison with the pollinated WT fruit.

## Discussion

Flower is an important organ for fruit production. The Rosaceae family contains several fruit crops of high economic value worldwide, such as apple, peach, pear, cherry, and strawberry. As most of them are woody trees, the genetic control of flower development is less studied in these species. ABCE homeotic genes play important roles in floral organ specification. In this study, different alleles of the E class gene *FveSEP3* were generated in *F. vesca* by EMS mutagenesis and CRISPR/Cas9. Taking advantage of these valuable mutants, we revealed that *FveSEP3* plays critical roles in both flower and fruit development.

### *FveSEP3* plays predominant E functions in flower development among *FveSEP*s

The *F. vesca* genome contains four *SEP* genes based on sequence similarities^18^. According to the transcriptome data, *FveSEP3* and *FveSEP4* were more abundantly expressed than the other two genes (Fig. 3e). The *fvesep3*^*CR*^ mutants, in which the function of *FveSEP3* should have been completely abolished due to frameshift and greatly reduced expression, exhibited severe defects in floral organ specialization, suggesting a predominant role of *FveSEP3* in flower development compared with other *FveSEP*s. This differs from the functional redundancy of the *SEP*s in Arabidopsis^10,11^. The point mutation in the EMS mutant R27/*fvesep3* was able to convert all floral organs to leaf-like structures in the type II flowers (Fig. 1 and Supplementary Fig. S1), a stronger phenotype than the type I flowers and the *fvesep3*^*CR*^ mutants. This may imply that strawberry *SEP*s also possess redundant roles but to a lesser extent than Arabidopsis homologs.

The heterozygous R27/*fvesep3* flowers developed petals with serrated margins (Fig. 1), and the homozygous flowers were separated into two types, suggesting that the R27 allele of *FveSEP3* is semidominant or dosage-dependent. When *FveSEP3* was overexpressed in Arabidopsis, the WT version did not cause an obvious phenotype (Supplementary Fig. S4), which was different from the *AtSEP3-ox* plants showing early flowering with solitary flowers and the early termination of inflorescence^28^. However, the overexpression of the mutant version *FveSEP3*^*G27E*^ led to the conversion of petals and stamens to sepaloid organs, resembling the *sep1/2/3* triple or *sep1/2/3/4* quadruple mutants in Arabidopsis^10,11^. Based on these observations, we hypothesize that *fvesep3*^*R27*^ might be a dominant-negative allele. The *FveSEP3*^*G27E*^ mutation is located in the conserved MADS domain, which is responsible for DNA binding^29^. Consistently, FveSEP3^G27E^ preserved the interaction capacity with other ABCE class MADS-box proteins (Supplementary Fig. S6). A previous study found that the highly conserved arginine residue at position 3 contributes significantly to the binding specificity^30^. Our mutation occurred at another highly conserved amino acid G at position 27 (Fig. 3a)^31^. Taken together, FveSEP3^G27E^ is able to form a complex with other proteins, but the complex might have lost or reduced the DNA-binding capacity to downstream genes, thereby causing the dominant-negative effect.

In addition to regulating floral organ specialization, *SEP* genes also take part in the maintenance of floral meristem identity in other plant species. The cosuppression of the *SEP3* homolog *FBP2* (*Floral Binding Protein 2*) in petunia resulted in the development of ectopic inflorescences^32,33^. A lack of *SEP*s could induce the formation of secondary flowers in Arabidopsis^10^. The reduced expression of the *SEP1*-like gene *SlTM29* caused the growth of ectopic shoots^14^. In contrast, no change in floral meristem determinacy was observed in either R27 or *fvesep3*^*CR*^, indicating that strawberry *SEPs* may have lost this function.

The class B and C genes were significantly downregulated in the flower buds of *fvesep3* (Fig. 5a), consistent with the loss of petal and stamen identity. A previous study revealed that AtSEP3 could directly bind to the promoter regions of the B and C genes^27^. As functional evidence, the severe *AtSEP3-ox* lines elicited the homeotic transformation of sepals and inflorescence meristems into carpelloid structures, indicating the activation of class B and C functions^34^. It is possible that the class B and C genes in strawberry might also be directly regulated by FveSEP3. Alternatively, expression level changes of the A, B, and C genes in *fvesep3* might also be an indirect effect of the conversion of stamens and carpels to sepals.

The ABCE class of MADS-box proteins directly interacts with each other to form tetrameric complexes and then binds to two neighboring CArG-box motifs of downstream genes^5–8^. The strong interactions between FveSEP3 and all ABCE class MADS-box proteins (except for the weak interaction with FveAP3) examined by both yeast two-hybrid and split luciferase assays indicate that this mechanism is also conserved in strawberry (Fig. 5b–d).

### *FveSEP3* is essential for fruit growth and ripening

The *fvesep3* mutants obtained by either EMS mutagenesis or genome editing in *F. vesca* exhibited the development of parthenocarpic fruit, which provides convincing evidence on the *SEP* genes repressing fruit growth in strawberry. The three *FaSEP3* genes in cultivated strawberry were also highly expressed in the fruit receptacle (Supplementary Fig. S2b), a similar expression pattern to *FveSEP3*, suggesting their conserved functions in different strawberry species. Several lines of evidence highlight the class B and E genes as key repressors of fruit growth. For instance, the insertion of transposons in the *MdPI* intron resulted in the development of parthenocarpic fruit in apple germplasms^17^. In contrast, ectopic expression of *PI* inhibited fruit flesh tissue growth, resulting in fleshless, small berries in both apple and grape (*Vitis vinifera*)^35,36^. The downregulation of the *SEP1*-like gene *TM29* caused parthenocarpic fruit in tomato^14^. In apple, suppression of the *SEP1/2*-like *MADS8/9* resulted in varying degrees of reduction of cortex tissue, the flesh from hypanthium, indicating a positive role of *SEP1/2* in fruit growth^16^. In our results, the receptacles were not enlarged in the type II flowers of R27 (Supplementary Fig. S1), implying the presence of *FveSEP*s playing opposing roles to *FveSEP3* in fruit growth.

Transcriptome analysis revealed that a small fraction of upregulated genes were shared in the young fruit of *fvesep3* (16.9%) and pollinated WT (22.5%) compared with the unpollinated WT fruit (Fig. 7b), indicating quite different transcriptome landscapes. However, several similar GO terms were enriched among these two gene lists, suggesting that similar biological processes were employed for fruit growth. Auxin is the key hormone regulating fruit initiation in strawberry^12,13,37^. We found that a number of genes involved in auxin biosynthesis, transport, and signaling were differentially expressed, such as the *ARF* and *IAA* genes (Fig. 7d, e). *Expansin* (*EXP*) and *small auxin upregulated RNA* (*SAUR*) genes act downstream of auxin signaling to induce cell expansion^38,39^. We found several differentially expressed *EXP* and *SAUR* genes in the comparisons, and a majority of them were specific to only one sample (Data S1). Additionally, several GA pathway genes were also differentially expressed (Fig. 7d), consistent with the important roles of GA in early fruit development in strawberry^13^. A feature here is that different members in the same gene family were adopted by either *fvesep3* or pollinated WT fruit, which may explain the lower percentage of shared genes.

The expression level of *FveSEP3* was increased in fruit from the green to the turning stage (15D vs. 22D) according to the transcriptome data, suggesting a possible role of *FveSEP3* in fruit ripening (Fig. 3e). Indeed, fruit ripening in *fvesep3* was significantly delayed. The *SEP* genes in other clades are also responsible for fruit ripening in different species. For instance, silencing the *SEP1/2*-like gene *FaMADS9* in cultivated strawberry resulted in retarded fruit maturation^20^. Suppressed expression of the *SEP1/2*-like genes *PrupeSEP1* in peach and *PpMADS7* in sweet cherry by virus-induced gene silencing delayed fruit ripening and softening^40,41^. Similarly, reduced expression of the *SEP1/2*-like genes *MaMADS1* or *MaMADS2* showed delayed ripening and longer shelf life in banana^42^. The well-known *SEP1*-like gene *SlMADS-RIN* (*RIN*) in tomato has been identified as a master regulator of fruit ripening^43^. In addition, the class C homeotic gene *PrpPLENA* is upregulated during ripening in peach, and the overexpression of this gene in tomato accelerates fruit ripening^44^. These results indicate that floral homeotic genes are broadly involved in the regulation of fruit ripening. In the climacteric fruit tomato, *RIN* directly regulated the expression of key enzymes of ethylene biosynthesis during fruit ripening control^45^. PrpSEP1 can bind to the promoter of the cell wall softening enzyme polygalacturonase^40^. Strawberry fruit belongs to the no climacteric type of ripening that is primarily regulated by the ABA pathway^1^. How *FveSEP3* interacts with the ABA pathway or other associated genes during fruit ripening awaits further investigation.

## Materials and Methods

### Plant materials and growth conditions

Three *F. vesca* accessions, Ruegen (red fruited), Yellow Wonder, and Hawaii 4 (white fruited), were used as the WT in this study. Both woodland strawberry and Arabidopsis plants were grown in a growth room under a light intensity of 100 μmol m^−2^ s^−1^ under a photoperiod of 16 h light and 8 h dark at 22 °C.

### Gene isolation of the EMS mutant R27

Heterozygous R27 was crossed with WT Ruegen; F_1_ heterozygous R27 plants were selected and allowed to self-cross to generate an F_2_ population. Equal amounts of young leaves were pooled from 20 F_2_ homozygous and 20 F_2_ heterozygous mutants. Genomic DNA was extracted using a CTAB method. Genome sequencing was performed using the Illumina HiSeq X Ten platform (Novogene, Beijing) and analyzed as described previously^46^. The candidate mutation was examined among individual F_2_ mutants by cloning and Sanger sequencing.

### Scanning electron microscopy

Samples were fixed in 2.5% glutaraldehyde at 4 °C overnight; washed with phosphate-buffered saline (PBS; 0.1 M, without NaCl) 3–5 times (15 min each time); fixed for 2–3 h in osmium acid; washed with PBS (0.1 M, without NaCl) 3–5 times (15 min each time); treated sequentially with 30, 50, 70, 80, 90, 95, and 100% ethanol for 15 min each; transferred to isoamyl acetate 3 times (20 min each time); critical point dried; coated with gold for 45 s; and photographed under a scanning electron microscope (JSM-6390LV).

### Phylogenetic analysis

The protein sequences were obtained from GDR for *F. vesca* (rosaceae.org), TAIR for Arabidopsis (Arabidopsis.org), and Sol Genomics Network for tomato (solgenomics.net). An unrooted phylogenetic tree was constructed using MEGA7 with the neighbor-joining statistical method and bootstrap analysis (1000 replicates).

### Plasmid construction

Genomic DNA or cDNA derived from RNA extracted from flowers or young leaves of Ruegen or R27 was used for sequence amplification. For overexpression, the full-length coding sequences of *FveSEP3* and *FveSEP3*^*G27E*^ were cloned into pENTR1A and inserted into the binary vector pK7WG2D. For genome editing, two *FveSEP3*-specific sgRNAs were designed using the web server CRISPR-P2.0 (http://crispr.hzau.edu.cn/CRISPR2/). Two AtU6 promoter-sgRNA-AtU6 terminator cassettes were amplified by PCR using pCBC-DT1T2 as the template and then inserted into pHSE401G^47^ by Golden Gate Assembly and confirmed by Sanger sequencing. For subcellular localization, the coding sequence of *FveSEP3* was cloned into the binary vector pRI101 at the *Eco*RI and *Nde*I sites and fused with *GFP* by the Gibson cloning method. The primers are shown in Table S4.

### Stable transformation in woodland strawberry

Woodland strawberry transformation was carried out as described previously^48^. The CRISPR/Cas9 construct was transformed into the *F. vesca* variety H4. During transformation, positive transgenic calli and regenerated plants were selected using both hygromycin (4 mg l^−1^) and GFP fluorescence, which was examined under a fluorescence dissecting microscope (Microshot Technology Ltd, Guangzhou, China, MZX81).

### Stable transformation in *Arabidopsis*

Arabidopsis Col-0 was transformed with *FveSEP3-ox* and *FveSEP3*^*G27E*^*-ox* in *Agrobacterium tumefaciens* GV3101 using the floral-dip method. The T_1_ transgenic lines were selected on half-strength MS (M5524, Sigma-Aldrich) with 100 mg l^−1^ kanamycin.

### Subcellular localization analysis

Agrobacterium colonies of *FveSEP3-GFP* and the nuclear marker VirD2NLS-mCherry^49^ were grown in 2 ml of liquid LB medium at 28 °C overnight. The culture was then spun down and resuspended in buffer (5 g l^−1^ D-glucose, 50 mM MES, 2 mM Na_3_PO_4_, 100 μM acetosyringone, pH 5.6) to reach an OD600 of 0.3. Equal volumes of the two suspensions were mixed together and infiltrated into the leaves of 3-week-old tobacco (*Nicotiana benthamiana*) using syringes. After 2 days of infiltration, images of GFP fluorescence were taken using a confocal laser scanning microscope (TCS SP8; Leica Wetzlar, Germany).

### Transcriptome sequencing and data analysis

Total RNA was isolated from the entire fruit (achenes and receptacle) at 6–7 DPA. Each sample had three biological replicates. Sequencing was performed on an Illumina NovaSeq platform (Shanghai Personal Biotechnology Co., Ltd, China). The *F. vesca* reference genome with the ver4.0.a2 annotation^26^ was downloaded from the GDR website (www.rosaceae.org). Raw reads were first trimmed 9 bp at the 5′ end using Trimmomatic^50^ and then mapped to the reference genome using the program hisat2^51^. FeatureCounts^52^ was used to count the reads mapped to the gene models. Gene expression levels were calculated as the TPM (transcript per million reads) with Tbtools^53^. Differential gene expression analysis was implemented by the R package DESeq2^54^, and those with an adjusted *P* value of <0.05 and fold change >2 were considered differentially expressed. PCA was performed using the R package factoextra (ver1.0.7) based on log_2_-transformed TPM + 1. A Venn diagram was made at Venny 2.1 (https://bioinfogp.cnb.csic.es/tools/venny). Tbtools was used for the GO enrichment analysis. Heatmaps were drawn using MeV 4.9.0.

### Quantitative RT-PCR

Total RNA was isolated from different tissues using a HiPure Plant RNA Mini Kit (Magen, Guangzhou, China; cat no. R4151) and treated with DNase I (Promega) according to the manufacturer’s instructions. Approximately 1 μg of total RNA was used for cDNA synthesis using a PrimeScript^TM^ RT Reagent Kit (TaKaRa, Shiga, Japan; cat no. RR047A). For qPCR, a total volume of 10 μl reaction mixture was set up containing 5 μl of 2× SYBR Green PCR master mix (Catalog # 172–5124, BioRad), 1 μl of 5× diluted cDNA, 0.25 μl of each primer, and 3.5 μl ddH_2_O. Amplification was performed using a Quant Studio 7 Flex system (Applied Biosystems, Waltham, MA, USA). The expression level of each gene was calculated using the 2^−∆∆CT^ method. FvH4_1g05910, the homolog of CDC27 in the anaphase-promoting complex, and the actin gene At3G18780 were used as the internal controls. All analyses were repeated, and each sample included three biological replicates.

### Yeast two-hybrid assays

Yeast two-hybrid assays were performed using the Matchmaker^TM^ Gold Two-Hybrid System (Clontech) following the manufacturer’s instructions. The coding sequences of the woodland strawberry ABCE genes were amplified from the cDNA of young WT flowers and inserted into the BD vector pGBKT7 or the AD vector pGADT7. Combinations of BD and AD vectors were cotransformed into the yeast (*Saccharomyces cerevisiae*) strain AH109. The transformants were selected on SD-Leu-Trp plates (DDO). The interactions were tested on SD-Leu-Trp-His-Ade plates (QDO). At least six individual clones were analyzed.

### Split-luciferase assays

Full-length *FveSEP3* was cloned into the JW771-nLUC vector. The full-length ABCE genes were cloned into the JW772-cLUC vector. The constructs were transferred into *A. tumefaciens* strain GV3101, and different combinations at a ratio of 1:1 at an OD600 of 0.5 were coinfiltrated into tobacco (*N. benthamiana*) leaves. After 2 days, the tobacco leaves were sprayed with 100 mM luciferin, kept in the dark for 10 min, and observed under a low-light cooled charge-coupled device imaging apparatus (LB985 NightSHADE).

### Statistical analyses

Statistical analyses were performed using SPSS v22.0 (IBM Corp., Armonk, NY, USA). Pairwise comparisons were determined using Student’s *t* test (**, *P* < 0.01).

## Supplementary information

Supplementary figures and tables

Dataset 1

## Data Availability

Raw reads of the transcriptome data have been submitted to the Sequence Read Archive at NCBI under the accession number PRJNA717819. The loci of strawberry and Arabidopsis genes or accession numbers of the genes in other species in NCBI are as follows: gene04229/FvH4_6g46420 (*FveSEP1*), gene04563/FvH4_4g29610 (*F veSEP-Like*), gene07201/FvH4_4g23530 (*FveSEP3*), gene26118/FvH4_5g13510 (*FveSEP4*); Arabidopsis: AT5G15800 (*SEP1*), AT1G24260 (*SEP2*), AT1G24260 (*SEP3*), AT2G0371 (*SEP4*); *Solanum lycopersicum*: Solyc02g089200.2 (*TM29*), Solyc04g005320.2 (*MADS-box TF*), Solyc05g015750.2 (*TM5*), Solyc03g114840.2 (*MADS1*); *Malus domestica*: ACJ64679 (*MdMADS1*), AAD51422 (*MdMADS3*), ACJ64681 (*MdMADS4*), JN651403 (*MdMADS6*), ADL36749 (*MdMADS7*), CAA04919 (*MdMADS8*), ADL36750 (*MdMADS9*), ADL36752 (*MdMADS11*), ADL36737 (*MdMADS14*), CAC80858 (*MdMADS15*), ADL36740 (*MdMADS18*), ADL36747 (*MdMADS24*).
