# Supplementary material for: The MADS-box gene FveSEP3 plays essential roles in flower organogenesis and fruit development in woodland strawberry
Source: Hortic Res. 2021 Dec 1;8:247. doi: 10.1038/s41438-021-00673-1 (PMC8632884; doi:10.1038/s41438-021-00673-1)
Supplement: Supplementary file 1 — Supplementary figures and tables [file 41438_2021_673_MOESM1_ESM.docx]

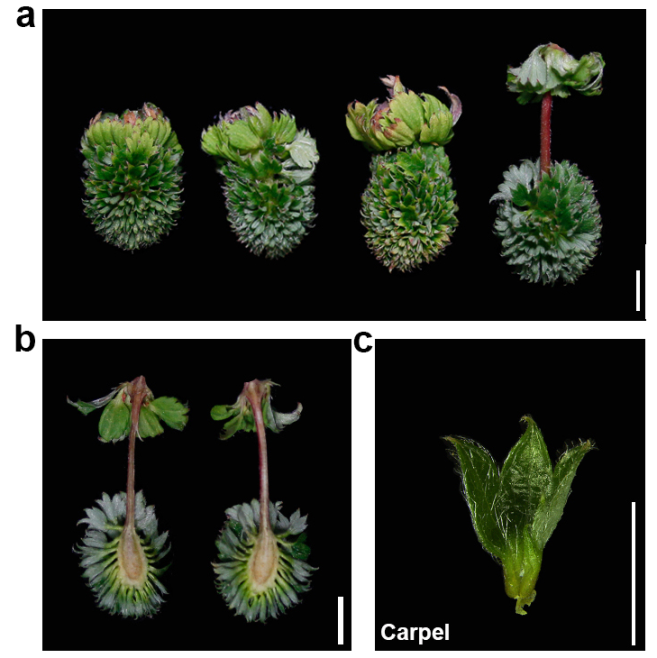


**Supplementary Figure S1. R27-type II flowers.**

**a** Images of R27-type II flowers at 30 DPA (days after anthesis). **b** Longitudinal sections of R27-type II flowers at 30 DPA. **c** One carpel obtained from the R27-type II flower. Scale bars: 0.5 cm.


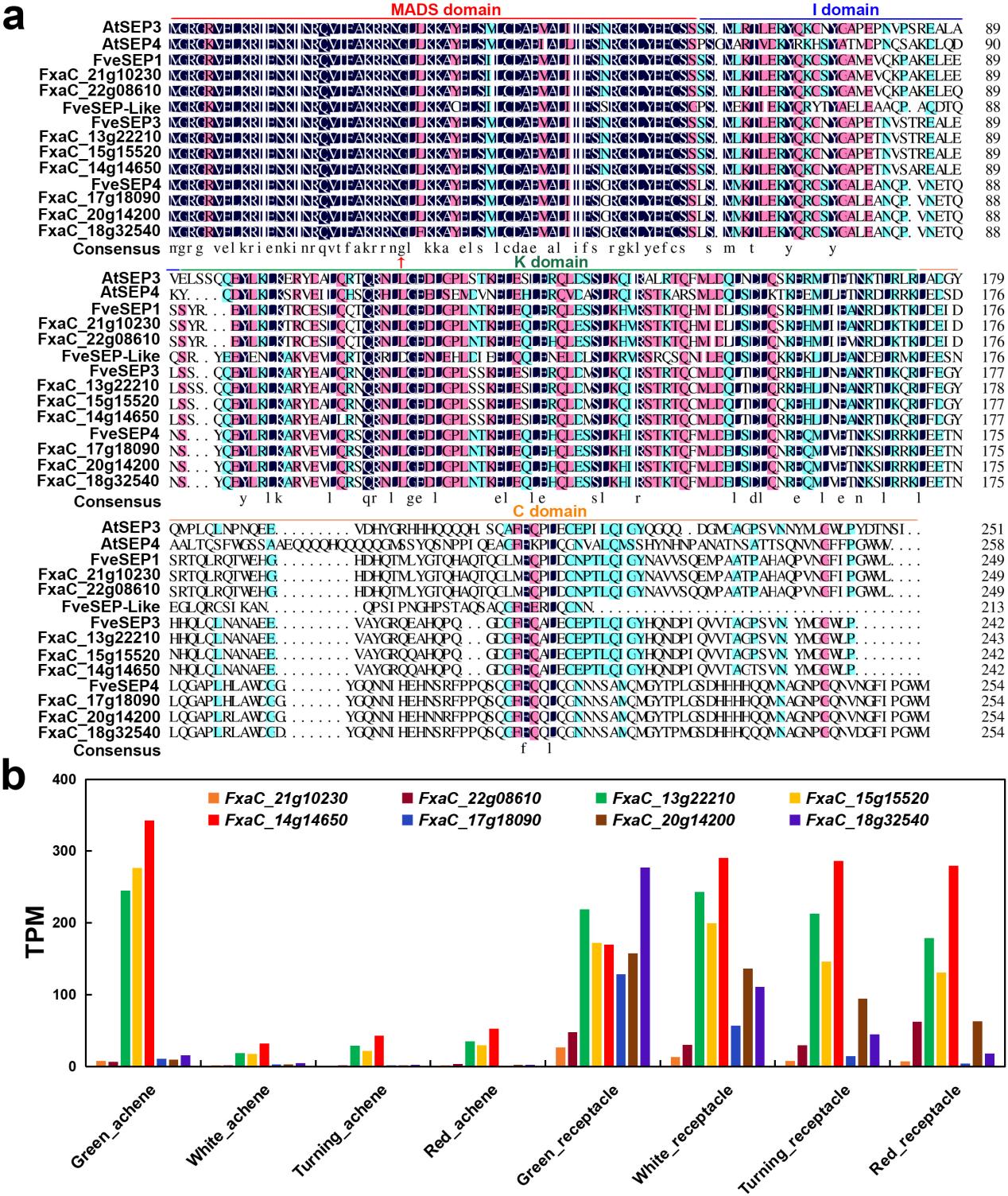


**Supplementary Figure S2. Protein sequence alignment and expression patterns of *SEP* genes in cultivated strawberry.**

**a** Protein sequence alignment of SEPs from Arabidopsis, *F. vesca*, and cultivated strawberry (*Fragaria x ananassa*; genes are labeled as FxaC_XXgXXXXX). **b** The expression patterns of *FaSEPs* in the fruit of cultivated strawberry according to RNA-seq data (Liu *et al.*, Horticulture Research, 2021). Four developmental stages (green, white, turning, and red) in two tissue types (achene and receptacle) are included. The expression levels are indicated by TPM.


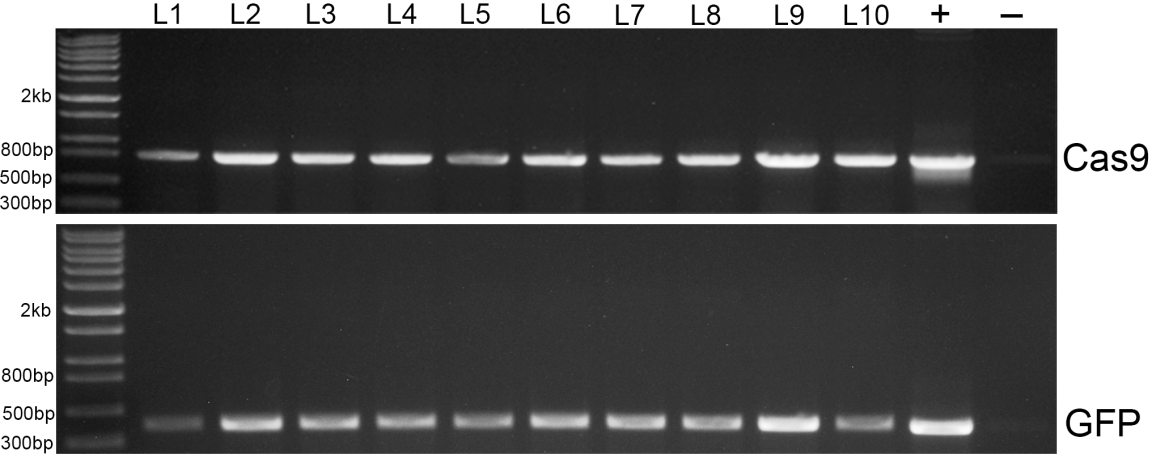


**Supplementary Figure S3. Genotypes of the *FveSEP3-CR* transgenic lines in *F. vesca*.**

Gel electrophoresis images showing the amplified bands of *Cas9* and *GFP* from 10 independent transgenic lines in the T_0_ generation. The plasmid is used as the positive control, and the wild-type *F. vesca* is used as the negative control.


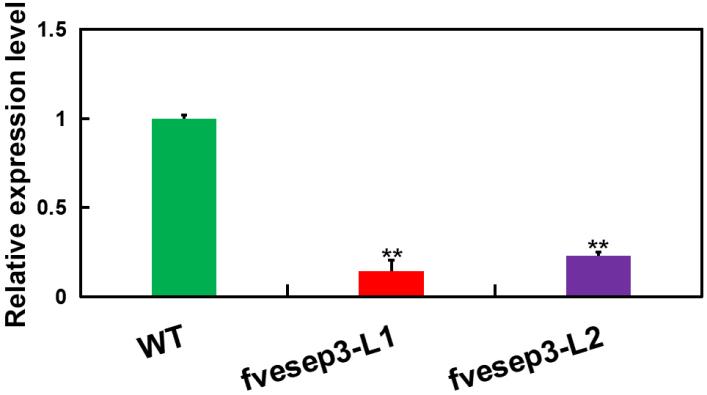


**Supplementary Figure S4. The expression levels of *FveSEP3* in the *fvesep3^CR^* mutants.**

Bar plot showing the expression levels of *FveSEP3* in fruit at 6–7 DPA of the two *fvesep3^CR^* mutants (L1 and L2), as examined by qRT-PCR. Data are the means±SD obtained from three technical replicates. **, *P* < 0.01, Student’s *t*-test.


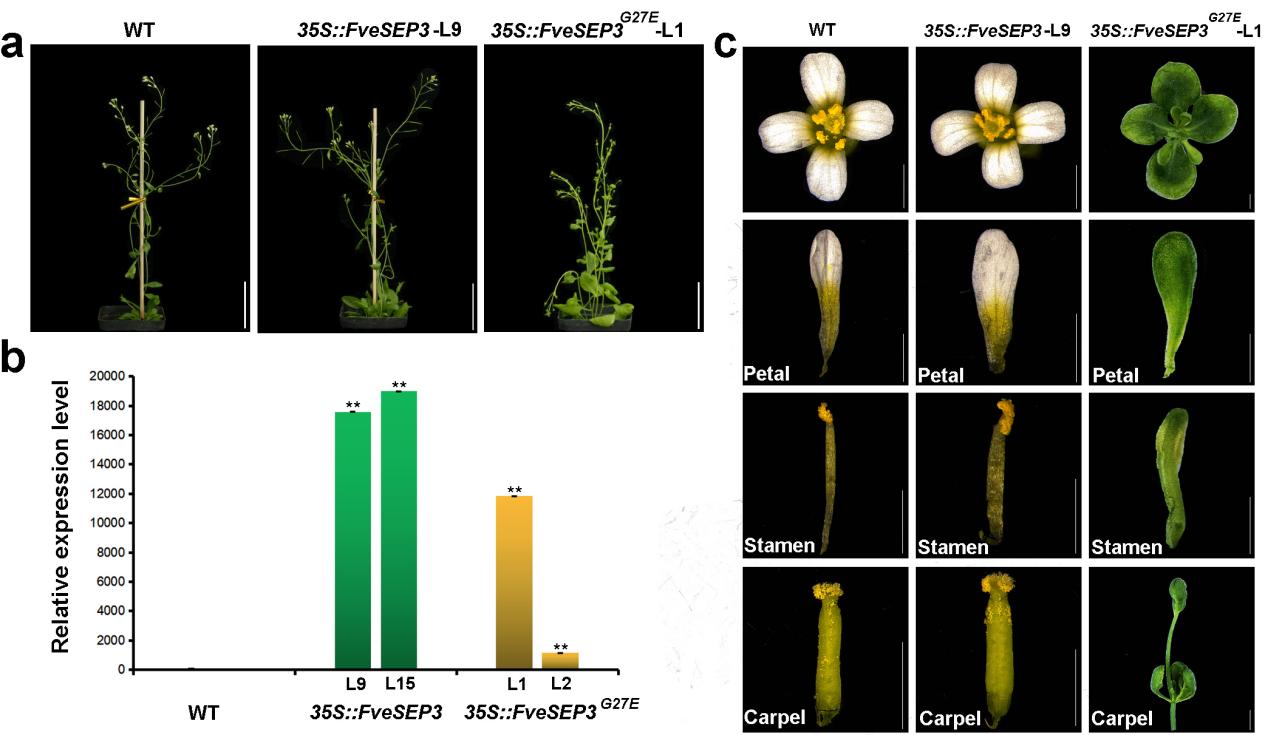


**Supplementary Figure S5. Phenotypes of the *FveSEP3-ox* and *FveSEP3^G27E^-ox* transgenic lines in Arabidopsis.**

**a** Plants of *FveSEP3-ox* and *FveSEP3^G27E^-ox* transgenic Arabidopsis in the Col-0 background. Scale bars: 5 cm. **b** The expression levels of *FveSEP3* or *FveSEP3^G27E^* in the flowers of wild-type and two independent transgenic lines in the T_1_ generation, as analyzed by qRT-PCR. Data are the means±SD obtained from three technical replicates. The values of two independent transgenic lines are compared to wild-type: **, *P* < 0.01, Student’s *t*-test. **c** Flowers of the *FveSEP3-ox* and *FveSEP3^G27E^-ox* transgenic Arabidopsis in the Col-0 background. Each panel from top to bottom shows entire flowers, petals, stamens, and carpels. Scale bars: 0.5 mm.

**
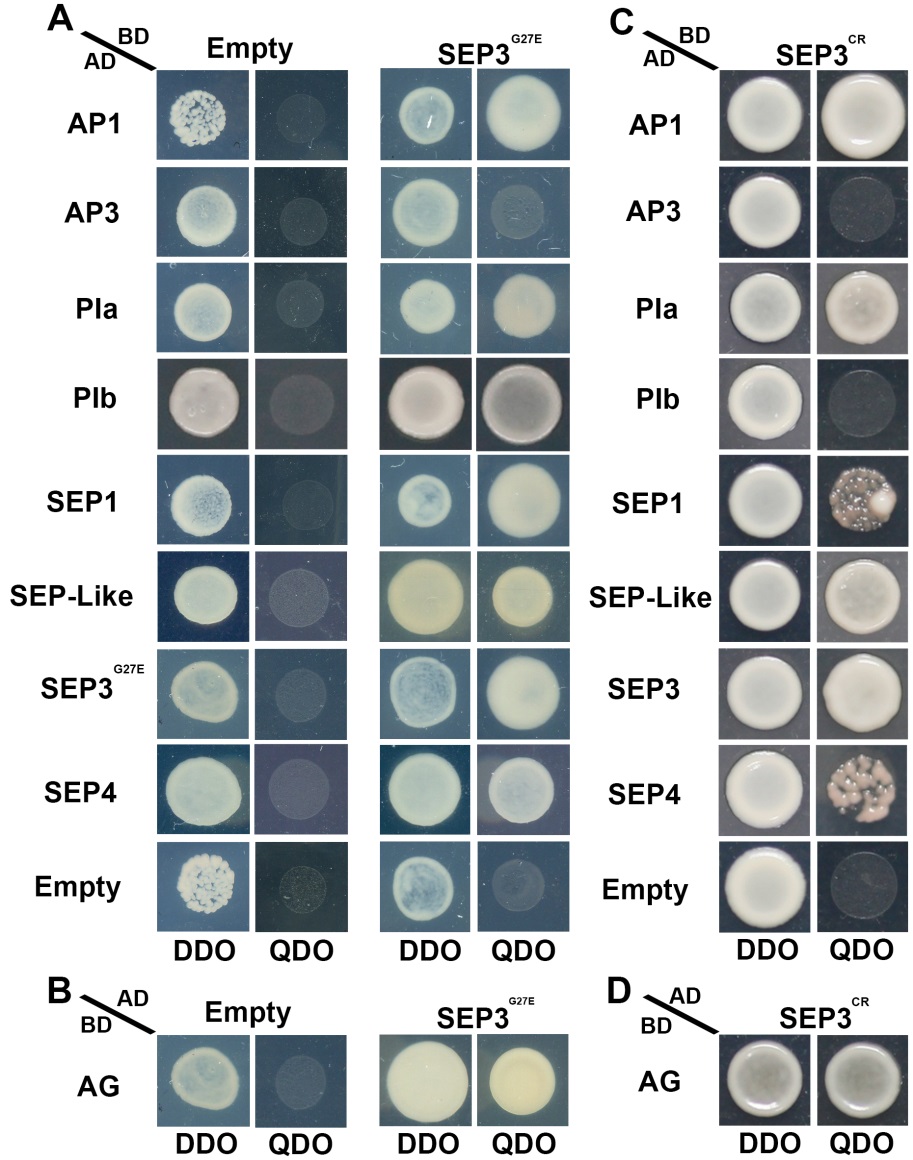
**

**Supplementary Figure S6. Physical interactions between mutated FveSEP3 and ABCE class MADS box proteins.**

(**a**, **b**) Physical interactions between FveSEP3^G27E^ and class ABCE proteins in strawberry, as examined by a yeast two-hybrid assay. (**c**, **d**) Physical interactions between FveSEP3^CR^ and ABCE class MADS box proteins in strawberry, as examined by a yeast two-hybrid assay. Transformed yeast cells were grown on SD-Leu-Trp (DDO) and SD-Leu-Trp-His-Ade (QDO). AD, activation domain; BD, DNA-binding domain. Empty vectors were used as controls.

**
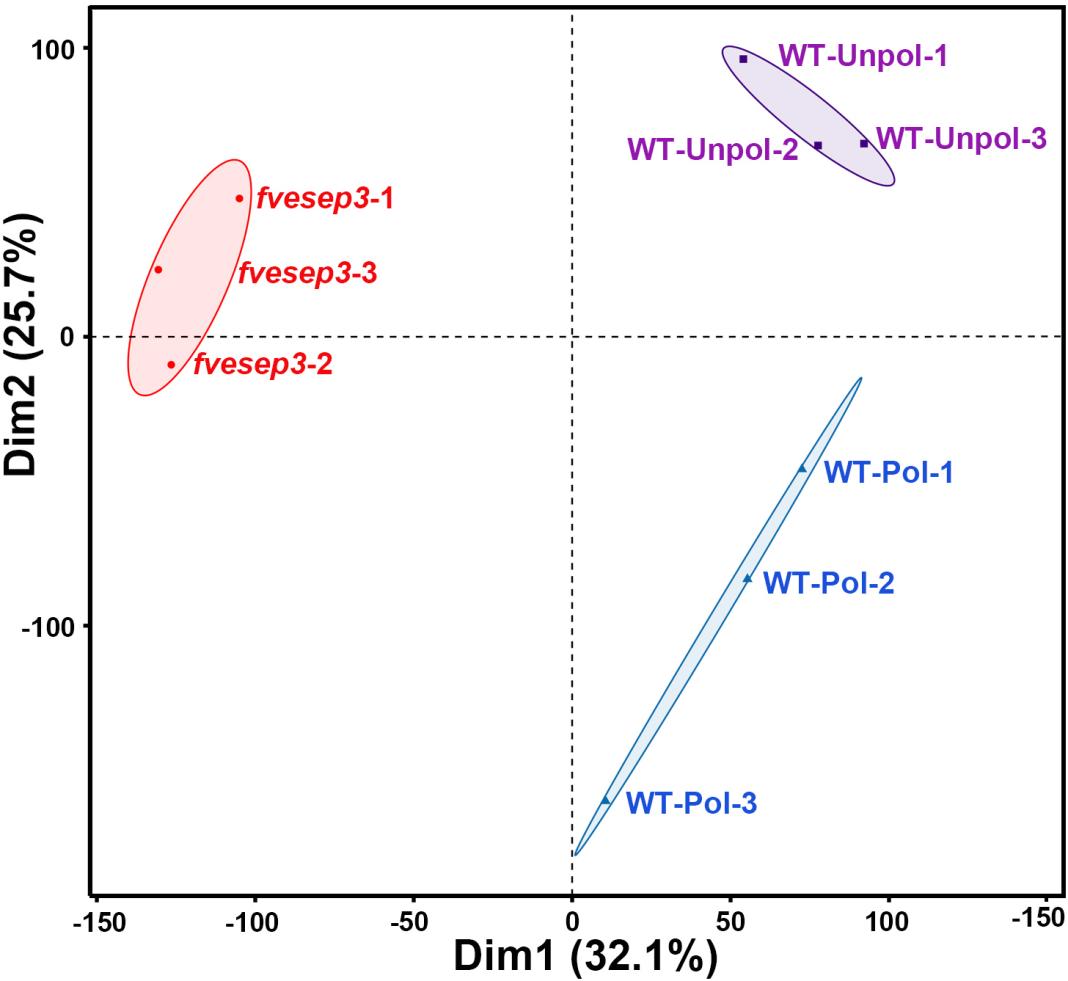
**

**Supplementary Figure S7. Principal component analysis (PCA) of the RNA-seq samples.**

The log_2_-transformed TPM values of all the expressed genes were used for the PCA analysis. The fruit at 6–7 DPA of *fvesep3*, WT-Pol, and WT-Unpol with three biological replicates were examined. Ellipse indicates the 95% confidence area.

**Table S1. The list of candidate SNPs in the EMS mutant R27.**

| **Chromosome** | **Position** | **DNA change** | **Amino acid change** | **%SNP in**  **mutant library** | **Gene ID (v2.0.a2)** | **Gene ID (v4.0.a2)** |
| --- | --- | --- | --- | --- | --- | --- |
| Fvb3  Fvb4  Fvb4  Fvb4  Fvb4  Fvb4  Fvb4  Fvb4  Fvb4  Fvb4  Fvb4  Fvb4  Fvb4  Fvb4  Fvb4  Fvb4  Fvb4  Fvb4  Fvb4 | 28398168  5554601  7109267  9374029  9705273  13004108  14175371  15145166  17160591  17705215  22922177  23136338  236094820 | GAC-AAC | D-N | C:11% T:89% | gene28839 | FvH4_3g33040 |
| Fvb4 | 5554601 | AAG-AAA | K | A:93% G:8% | gene12321 |  |
| Fvb4 | 7109267 | GGC-GAC | G-D | C:6% T:94% | gene36597 | FvH4_4g07673 |
| Fvb4 | 9374029 | CCG-TCG | P-S | C:3% T:97% | gene21703 |  |
| Fvb4 | 9705273 | GTC-GTT | V | A:100% | gene29640 |  |
| Fvb4 | 13004108 | GGC-GGT | G | T:100% | gene27656 | FvH4_4g08820 |
| Fvb4 | 14175371 | GGC-GGT | G | C:4% T:96% | gene22387 | FvH4_4g11622 |
| Fvb4 | 15145166 | CCT-CTT | P-L | A:95% G:5% | gene06608 | FvH4_4g12942 |
| Fvb4 | 17160591 | GCC-ACC | A-T | A:97% G:3% | gene36679 | FvH4_4g15110 |
| Fvb4 | 17705215 | GAC-AAC | D-N | C:3% T:97% | gene09777 | FvH4_4g15620 |
| Fvb4 | 22922177 | GTA-ATA | V-I | A:97% G:3% | gene36740 | FvH4_4g20990 |
| Fvb4 | 23136338 | GGC-AGC | G-S | A:100% | gene22974 | FvH4_4g21340 |
| Fvb4 | 23609482 | TGG-TAG | W-STOP | A:32% G:68% | gene22766 | FvH4_4g21881 |
| Fvb4 | 24150589 | AGG-AGA | R | C:2% T:98% | gene7017 | FvH4_4g25380 |
| Fvb4 | 24196711 | TCT-TTT | S-F | C:5% T:95% | gene07025 | FvH4_4g25290 |
| Fvb4 | 25299357 | GGA-GAA | G-E | A:100% | gene07201 | FvH4_4g23530 |
| Fvb4 | 26427808 | GAT-AAT | D-N | A:98% G:2% | gene03622 | FvH4_4g26261 |
| Fvb4 | 27123505 | ATG-ATA | V-I | A:95% G:5% | gene03747 | FvH4_4g27430 |
| Fvb4 | 27295700 | CCT-TCT | P-S | A:100% | gene03779 | FvH4_4g27770 |

**Table S2. Summary of RNA-seq read statistics.­**

| **SampleID** | **Number of raw reads** | **Number of uniquely mapped reads** | **% mapped** |
| --- | --- | --- | --- |
| *fvesep3*-1 | 32,126,410 | 31,059,230 | 96.68% |
| *fvesep3*-2 | 35,735,028 | 34,554,709 | 96.70% |
| *fvesep3*-3 | 35,218,553 | 34,110,998 | 96.86% |
| WT-Unpol-1 | 33,993,508 | 32,352,951 | 95.17% |
| WT-Unpol-2 | 31,716,669 | 30,324,117 | 95.61% |
| WT-Unpol-3 | 29,444,875 | 28,162,197 | 95.64% |
| WT-Pol-1 | 33,603,133 | 31,882,581 | 94.88% |
| WT-Pol-2 | 43,394,320 | 41,843,541 | 96.43% |
| WT-Pol-3 | 36,139,647 | 34,682,671 | 95.97% |
| Average |  |  | 95.99% |

**Table S3. The expression levels (TPM) of auxin genes shown in the heatmap.**

| **Gene** | **WT-Pol-1** | **WT-Pol-2** | **WT-Pol-3** | **WT-Unpol-1** | **WT-Unpol-2** | **WT-Unpol-3** | ***fvesep3*-1** | ***fvesep3*-2** | ***fvesep3*-3** | **GeneID (v2.0.a2)** | **GeneID (v4.0.a2)** |
| --- | --- | --- | --- | --- | --- | --- | --- | --- | --- | --- | --- |
| FveYUC10 | 18.38 | 24.89 | 29.18 | 12.07 | 13.39 | 13.21 | 103.80 | 103.42 | 88.65 | gene27796 | FvH4_2g24750 |
| FvePIN5 | 5.35 | 3.62 | 7.32 | 3.02 | 7.98 | 5.59 | 11.56 | 13.86 | 16.51 | gene16792 | FvH4_6g00660 |
| FvePIN10 | 2.90 | 4.48 | 13.25 | 0.68 | 0.45 | 0.70 | 2.51 | 1.91 | 0.34 | gene12312 | FvH4_4g06850 |
| FveLAX1 | 96.98 | 79.61 | 131.02 | 45.92 | 56.14 | 63.01 | 207.66 | 228.70 | 135.61 | gene20938 | FvH4_7g24200 |
| FveLAX2 | 181.78 | 188.34 | 131.69 | 348.25 | 249.72 | 252.35 | 60.61 | 56.57 | 84.90 | gene11123 | FvH4_2g26410 |
| FveGH3.1 | 12.86 | 22.87 | 41.56 | 14.59 | 11.55 | 43.22 | 5.55 | 6.32 | 2.63 | gene22838 | FvH4_2g04750 |
| FveGH3.17 | 18.42 | 25.33 | 46.10 | 4.00 | 3.14 | 4.39 | 14.47 | 15.40 | 21.58 | gene23026 | FvH4_4g22430 |
| FveGH3.18 | 6.15 | 4.56 | 5.16 | 3.37 | 4.71 | 5.26 | 42.46 | 51.21 | 30.73 | gene10286 | FvH4_6g44990 |
| FveGH3.5 | 6.64 | 4.72 | 6.93 | 12.01 | 9.94 | 5.84 | 0.81 | 1.51 | 0.98 | gene03265 | FvH4_3g21460 |
| FveARF2 | 39.16 | 35.00 | 21.40 | 113.89 | 79.13 | 97.16 | 10.45 | 12.02 | 9.42 | gene08492 | FvH4_2g38760 |
| FveARF7 | 8.14 | 8.51 | 12.01 | 5.26 | 5.13 | 5.58 | 17.41 | 16.86 | 14.30 | gene16844 | FvH4_3g32000 |
| FveARF13 | 51.72 | 52.33 | 59.57 | 29.22 | 38.05 | 34.94 | 24.68 | 20.32 | 23.26 | gene28545 | FvH4_3g32860 |
| FveIAA4 | 62.64 | 92.93 | 99.85 | 26.29 | 43.10 | 44.10 | 42.46 | 69.18 | 36.20 | gene05993 | FvH4_6g30850 |
| FveIAA6 | 3.03 | 3.90 | 10.99 | 2.07 | 2.11 | 2.29 | 6.67 | 16.86 | 6.10 | gene08191 | FvH4_2g22530 |
| FveIAA7 | 91.50 | 118.37 | 145.68 | 33.25 | 44.86 | 50.64 | 33.17 | 44.20 | 30.01 | gene08336 | FvH4_2g20500 |
| FveIAA20 | 93.33 | 100.91 | 56.17 | 172.70 | 141.79 | 150.82 | 322.81 | 639.63 | 377.79 | gene08194 | FvH4_2g22520 |
| FveIAA21 | 51.18 | 61.90 | 77.30 | 24.44 | 27.60 | 24.81 | 31.44 | 23.58 | 20.12 | gene27891 | FvH4_4g04700 |
| FveIAA24 | 26.09 | 35.96 | 45.76 | 7.61 | 12.20 | 12.56 | 13.89 | 12.20 | 12.67 | gene22779 | FvH4_4g21780 |
| FveIAA26 | 35.18 | 46.25 | 62.49 | 6.27 | 13.74 | 15.37 | 6.65 | 14.57 | 1.69 | gene05990 | FvH4_6g30860 |
| FveGA2ox2 | 3.84 | 7.66 | 5.19 | 7.95 | 6.57 | 17.47 | 0.42 | 0.60 | 0.73 | gene00852 | FvH4_4g36510 |
| FveGA2ox4 | 8.54 | 8.62 | 13.28 | 4.74 | 6.77 | 5.21 | 11.99 | 14.59 | 17.10 | gene07935 | FvH4_3g38920 |
| FveGA3ox1 | 4.70 | 5.51 | 6.20 | 19.52 | 8.75 | 6.85 | 1.77 | 0.97 | 0.42 | gene06004 | FvH4_6g30780 |
| FveGA3ox5 | 1.40 | 4.34 | 12.39 | 0.78 | 0.07 | 0.41 | 0.57 | 0.54 | 0.69 | gene01060 | FvH4_2g30010 |
| FveGA3ox6 | 0.72 | 4.69 | 16.30 | 0.00 | 0.00 | 0.00 | 0.10 | 0.16 | 0.19 | gene11192 | FvH4_2g27140 |
| FveSNE | 7.27 | 4.81 | 5.65 | 3.28 | 9.29 | 6.74 | 0.47 | 1.71 | 0.99 | gene15587 | FvH4_6g40870 |
| FveGID1b | 169.87 | 134.82 | 35.33 | 363.08 | 379.37 | 329.64 | 82.25 | 64.84 | 83.11 | gene27756 | FvH4_2g24370 |
| FveKS1 | 4.67 | 5.95 | 4.53 | 6.42 | 6.43 | 4.53 | 13.24 | 12.60 | 11.53 | gene19699 | FvH4_3g14116 |

**Table S4. Primers used in this study.**

| **Primer name** | **Sequence (5'-3')** | |
| --- | --- | --- |
| **Primers used for genotyping of the** *fvesep3* **mutant R27** | | |
| SEP3_F | TGGGGTTGGGTTAGTGAGGAAAGT | |
| SEP3_R | TGATCATGCCGCTAGCATAGCC | |
| **Primers used for making the FveSEP3-CRISPR construct** | | |
| FveSEP3-DT1-BsF | ATATATGGTCTCGATTGGAGACAAATGTATCTACAAGTT | |
| FveSEP3-DT1-F0 | TGGAGACAAATGTATCTACAAGTTTTAGAGCTAGAAATAGC | |
| FveSEP3-DT2-R0 | AACTTCGCTGAAGATCTGTGAGCAATCTCTTAGTCGACTCTAC | |
| FveSEP3-DT2-BsR | ATTATTGGTCTCGAAACTTCGCTGAAGATCTGTGAGCAA | |
| **Primers used for genotyping of the *fvesep3^CR^* mutants** | | |
| FveSEP3^CR^-sgRNA1-Test-F | | CGCTCTGATCTGACTTGATGTGAA |
| FveSEP3^CR^-sgRNA1-Test-R | | GGGCAACTTTCTGTTCAGGGTA |
| FveSEP3^CR^-sgRNA2-Test-F | | TGCAGTGGTCTTCCTAGTGGAT |
| FveSEP3^CR^-sgRNA2-Test-R | | GTTTCAGTGTCCTGTTTGCCTCGT |
| **Primers used for making the *FveSEP3-ox* constructs** | | |
| 35S::FveSEP3-F | TCTTCACTGTTGATACATATGATGGGGAGGGGGAGAGTAGAGCTG | |
| 35S::FveSEP3-R | GCCCTTGCTCACCATGAATTCTGGCAACCATCCTCCCATGTAATT | |
| **Primers used for making the constructs in yeast two hybrid** | | |
| AP1-YHAD-F | GTACCAGATTACGCTCATATGATGGGAAGGGGTAGGGTTCAGCTG | |
| AP1-YHAD-R | ATGCCCACCCGGGTGGAATTCTGAAGCAAAGCATCCAAGGTGGCA | |
| AP3-YHAD-F | GTACCAGATTACGCTCATATGATGGAGTCAATCGAGTCCACTGTT | |
| AP3-YHAD-R | ATGCCCACCCGGGTGGAATTCTTTTTCAAATATGCATATTCCGAG | |
| PIa-YHAD-F | GTACCAGATTACGCTCATATGATGGGAAGGGGTAAGATTGAGATC | |
| PIa-YHAD-R | ATGCCCACCCGGGTGGAATTCGCAGTCGTGGTGGAGATTAGGCTG | |
| PIb-YHAD-F | GTACCAGATTACGCTCATATGATGGGGAGGGGTAAGATTGAGATT | |
| PIb-YHAD-R | ATGCCCACCCGGGTGGAATTCCATTATGTCGTGGAGATTGGGCTG | |
| AG-YHAD-F | GTACCAGATTACGCTCATATGATGGCCTATGAAAACAAACCAAAC | |
| AG-YHAD-R | ATGCCCACCCGGGTGGAATTCAACTAACTGAAGGGAAACTTGGTC | |
| SEP1-YHAD-F | GTACCAGATTACGCTCATATGATGGGGAGGGGAAGAGTGGAGCTG | |
| SEP1-YHAD-R | ATGCCCACCCGGGTGGAATTCGAGCATCCAACCAGGAATGAAACC | |
| SEP-Like-YHAD-F | GTACCAGATTACGCTCATATGATGGGAAGAGGGAAGGTGGAGCTG | |
| SEP-Like-YHAD-R | ATGCCCACCCGGGTGGAATTCGTTGTTGCATTGTAAACGCTCAAA | |
| SEP3-YHAD-F | GTACCAGATTACGCTCATATGATGGGGAGGGGGAGAGTAGAGCTG | |
| SEP3-YHAD-R | ATGCCCACCCGGGTGGAATTCTGGCAACCATCCTCCCATGTAATT | |
| SEP4-YHAD-F | GTACCAGATTACGCTCATATGATGGGGAGAGGGAGAGTTGAGCTC | |
| SEP4-YHAD-R | ATGCCCACCCGGGTGGAATTCAAGCATCCACCCAGGAATGAATCC | |
| AP1-YHBD-F | TCAGAGGAGGACCTGCATATGATGGGAAGGGGTAGGGTTCAGCTG | |
| AP1-YHBD-R | TCGACGGATCCCCGGGAATTCTGAAGCAAAGCATCCAAGGTGGCA | |
| AP3-YHBD-F | TCAGAGGAGGACCTGCATATGATGGAGTCAATCGAGTCCACTGTT | |
| AP3-YHBD-R | TCGACGGATCCCCGGGAATTCTTTTTCAAATATGCATATTCCGAG | |
| PIa-YHBD-F | TCAGAGGAGGACCTGCATATGATGGGAAGGGGTAAGATTGAGATC | |
| PIa-YHBD-R | TCGACGGATCCCCGGGAATTCGCAGTCGTGGTGGAGATTAGGCTG | |
| PIb-YHBD-F | TCAGAGGAGGACCTGCATATGATGGGGAGGGGTAAGATTGAGATT | |
| PIb-YHBD-R | TCGACGGATCCCCGGGAATTCCATTATGTCGTGGAGATTGGGCTG | |
| AG-YHBD-F | TCAGAGGAGGACCTGCATATGATGGCCTATGAAAACAAACCAAAC | |
| AG-YHBD-R | TCGACGGATCCCCGGGAATTCAACTAACTGAAGGGAAACTTGGTC | |
| SEP1-YHBD-F | TCAGAGGAGGACCTGCATATGATGGGGAGGGGAAGAGTGGAGCTG | |
| SEP1-YHBD-R | TCGACGGATCCCCGGGAATTCGAGCATCCAACCAGGAATGAAACC | |
| SEP-Like-YHBD-F | TCAGAGGAGGACCTGCATATGATGGGAAGAGGGAAGGTGGAGCTG | |
| SEP-Like-YHBD-R | TCGACGGATCCCCGGGAATTCGTTGTTGCATTGTAAACGCTCAAA | |
| SEP3-YHBD-F | TCAGAGGAGGACCTGCATATGATGGGGAGGGGGAGAGTAGAGCTG | |
| SEP3-YHBD-R | TCGACGGATCCCCGGGAATTCTGGCAACCATCCTCCCATGTAATT | |
| SEP4-YHBD-F | TCAGAGGAGGACCTGCATATGATGGGGAGAGGGAGAGTTGAGCTC | |
| SEP4-YHBD-R | TCGACGGATCCCCGGGAATTCAAGCATCCACCCAGGAATGAATCC | |
| **Primers used for making the constructs in split-luciferase assay** | | |
| SEP3-NLUC-F | ACGGGGGACGAGCTCGGTACCATGGGGAGGGGGAGAGTAGAGCTG | |
| SEP3-NLUC-R | CGCGTACGAGATCTGGTCGACTGGCAACCATCCTCCCATGTAATT | |
| AP1-CLUC-F | TACGCGTCCCGGGGCGGTACCATGGGAAGGGGTAGGGTTCAGCTG | |
| AP1-CLUC-R | ACGAAAGCTCTGCAGGTCGACTGAAGCAAAGCATCCAAGGTGGCA | |
| AP3-CLUC-F | TACGCGTCCCGGGGCGGTACCATGGAGTCAATCGAGTCCACTGTT | |
| AP3-CLUC-R | ACGAAAGCTCTGCAGGTCGACTTTTTCAAATATGCATATTCCGAG | |
| PIa-CLUC-F | TACGCGTCCCGGGGCGGTACCATGGGAAGGGGTAAGATTGAGATC | |
| PIa-CLUC-R | ACGAAAGCTCTGCAGGTCGACGCAGTCGTGGTGGAGATTAGGCTG | |
| PIb-CLUC-F | TACGCGTCCCGGGGCGGTACCATGGGGAGGGGTAAGATTGAGATT | |
| PIb-CLUC-R | ACGAAAGCTCTGCAGGTCGACCATTATGTCGTGGAGATTGGGCTG | |
| AG-CLUC-F | TACGCGTCCCGGGGCGGTACCATGGCCTATGAAAACAAACCAAAC | |
| AG-CLUC-R | ACGAAAGCTCTGCAGGTCGACAACTAACTGAAGGGAAACTTGGTC | |
| SEP1-CLUC-F | TACGCGTCCCGGGGCGGTACCATGGGGAGGGGAAGAGTGGAGCTG | |
| SEP1-CLUC-R | ACGAAAGCTCTGCAGGTCGACGAGCATCCAACCAGGAATGAAACC | |
| SEP-Like-CLUC-F | TACGCGTCCCGGGGCGGTACCATGGGAAGAGGGAAGGTGGAGCTG | |
| SEP-Like-CLUC-R | ACGAAAGCTCTGCAGGTCGACGTTGTTGCATTGTAAACGCTCAAA | |
| SEP3-CLUC-F | TACGCGTCCCGGGGCGGTACCATGGGGAGGGGGAGAGTAGAGCTG | |
| SEP3-CLUC-R | ACGAAAGCTCTGCAGGTCGACTGGCAACCATCCTCCCATGTAATT | |
| SEP4-CLUC-F | TACGCGTCCCGGGGCGGTACCATGGGGAGAGGGAGAGTTGAGCTC | |
| SEP4-CLUC-R | ACGAAAGCTCTGCAGGTCGACAAGCATCCACCCAGGAATGAATCC | |
| **Primers used for qRT-PCR** | | |
| AP1-qRT-F: | AATGTAGCGGAAGCTCAGGA | |
| AP1-qRT-R | ACGATGGCTCGAGAGTAAGG | |
| AP2-qRT-F | GGCCTATGACAAAGCTGCAA | |
| AP2-qRT-R | GTCCCAAATGCTGGGTTGTT | |
| AP3-qRT-F | GGGTATGAGGAGCAGAACGA | |
| AP3-qRT-R | TTGGAGCCCTCCCTTTGTAG | |
| PIa-qRT-F | TGCAGCGGCTCTCAAGAAAC | |
| PIa-qRT-R | GATGCCTGAGATGGACTTGC | |
| PIb-qRT-F | GCACTTGAAATTGGCCTTGC | |
| PIb-qRT-R | TGGGTGGTGGTGTTGTAGTT | |
| AG-qRT-F | GCTCTGTGATGCTGAGGTTG | |
| AG-qRT-R | CTTGGCAGCTTCTTGCTGAT | |
| SEP1-qRT-F | TTCTCTGTGACGCTGAGGTT | |
| SEP1-qRT-R | CACGATAGCTGCTCTCCTCA | |
| SEP-Like-qRT-F | TGTCGTTGAAGCGAGTTAGG | |
| SEP-Like-qRT-R | AGATGGGTGGCCATTTGGTA | |
| SEP3-qRT-F | AAGGGAGGCCTTGGAATTGA | |
| SEP3-qRT-R | GTGATTCGAGCTCCTTGCTG | |
| SEP4-qRT-F | CTGTGATGCTGAGGTTGCTC | |
| SEP4-qRT-R | GAGGACCTCCACTCTTGCTT | |
